# Subarachnoid Hemorrhage Following Ischemic Stroke Caused by Atrial Myxoma

**DOI:** 10.7759/cureus.17402

**Published:** 2021-08-23

**Authors:** Chao Chen, Yiya Xu, Wenjin Zhuang, Zhenhua Zhao, Yinzhou Wang

**Affiliations:** 1 Department of Neurology, Fujian Provincial Hospital, Fuzhou, CHN; 2 Shengli Clinical Medical College, Fujian Medical University, Fuzhou, CHN

**Keywords:** myxoma, subarachnoid hemorrhage, aneurysm, angiography, stroke, embolism

## Abstract

Subarachnoid hemorrhage (SAH) is a rare neurological complication of cardiac myxoma and is associated with poor outcomes. Previous reports have shown that myxoma-associated SAH was contributed by rupture of myxomatous intracerebral aneurysm. Here, we present an unusual case of angiographic-negative SAH in a young patient with left atrial myxoma.

A 28-year-old male was admitted for SAH. He had a history of magnetic resonance imaging (MRI)-confirmed ischemic stroke one year ago. The digital subtraction angiography (DSA) performed on next day revealed no intracerebral aneurysm or vascular malformation. Transthoracic echocardiography (TTE) showed a left atrial mass measuring 5.09 * 3.34 cm, indicating a diagnosis of atrial myxoma, which was confirmed by pathological examination. The cardiac tumor was excised and the patient’s symptoms improved completely. No intracerebral aneurysm was found by brain computed tomographic angiography (CTA) performed on day 24 after onset and one year after discharge. The patient remained asymptomatic during the one-year following-up.

The result suggests that SAH may be more commonly associated with cardiac myxoma than previously expected. And, mechanisms other than rupture of myxomatous intracerebral aneurysm involve in SAH associated with cardiac myxoma. Prolonged length of following-up using novel imaging technique should be applied to identify and monitor the change of source bleeding.

## Introduction

Cardiac myxoma accounts for 50-80% of primary heart tumors, and is important, though relatively uncommon, cause of cerebrovascular diseases [1]. The neurological complication occurs in more than 50% of myxoma patients and can be the initial manifestation [2]. The most frequent neurological complication of cardiac myxoma is cerebral embolism [3,4]. Intracerebral hemorrhage including parenchymal hematoma and subarachnoid hemorrhage (SAH) is less common in myxoma patients but usually leads to a much worse outcome than cerebral embolism. Rupture of myxomatous aneurysm is thought to be the major cause for both parenchymal hematoma and SAH associated with cardiac myxoma [5-9]. Myxoma complicated by SAH, with the absence of a myxomatous intracerebral aneurysm, is not reported. Here, we present a case of a young patient with SAH, 12 months after a preceded cerebral embolism. The SAH and cerebral embolism are demonstrated to be attributed to left atrial myxoma. But no aneurysm was found by multiple cerebral angiography. The result indicates an alternative mechanism for SAH associated with cardiac myxoma, and the risk of severe hemorrhagic complication of cardiac myxoma may be much higher than previously expected.

## Case presentation

**Figure 1:**
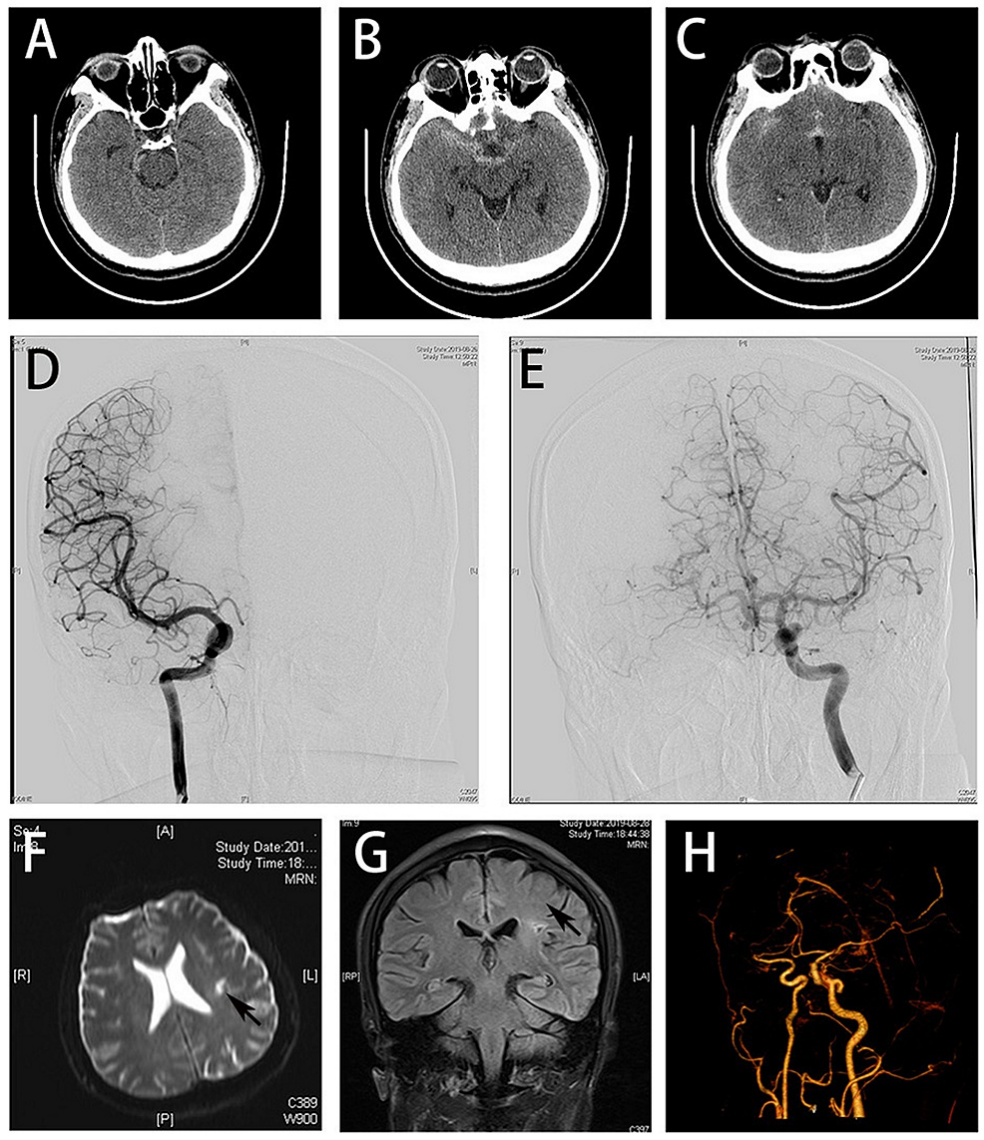
Multimodal neuroimaging of the patient (A-C) The emergent brain CT showed diffused SAH. (D and E) DSA on day two revealed no aneurysm. (F and G) Brain MRI showed previous stroke in left frontal lobe (black arrow). (H) Cerebral and cervical CTA performed at discharge revealed no aneurysm. SHA: subarachnoid hemorrhage; DSA: digital subtraction angiography; CTA: computed tomographic angiography

A 28-year-old male was referred to the emergency department of our hospital presenting with severe headache that occurred five hours ago when he was driving a car. The headache was of sudden onset and associated with intermittent vomiting. Non-contrast head computed tomography (CT) scan performed in the local hospital showed diffused hyperdensity in anterior and posterior fissure, Sylvian fissure, and around the brainstem, indicating the diagnosis of subarachnoid hemorrhage (SAH) (Figures 1A-1C). Twelve months ago, the patient suffered right-sided weakness. Brain magnetic resonance imaging (MRI) performed in the other hospital demonstrated acute ischemic stroke in the left frontal lobe. The hemiparesis has recovered completely and he has been taking aspirin 100 mg per day ever since. The patient has no other remarkable past medical history.

**Figure 2:**
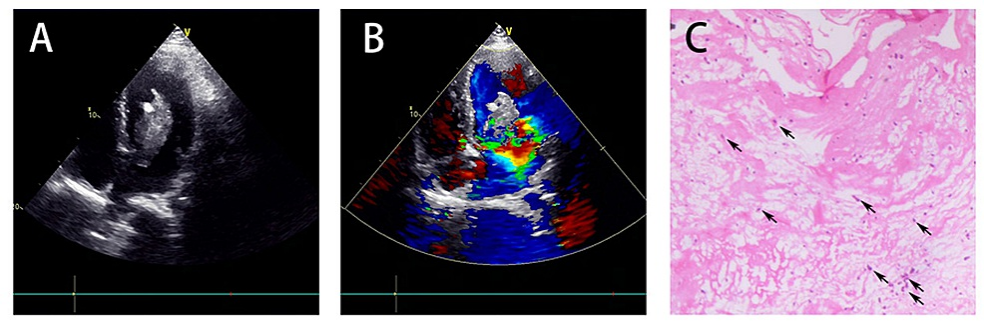
Echocardiographic and pathological findings (A and B) Echocardiography showed left atrial mass measuring 5.09 * 3.34 cm and moderate-to-severe mitral regurgitation. (C) Pathological examination of the mass tissue revealed stellate and spindle tumor cells (black arrow) surrounded by eosinophilic myxoid stroma.

The patient was admitted to the Department of Neurology for further management. On admission, he was afebrile and drowsy, with heart rate of 69 beats/min, respiration rate of 22 breaths/min, and arterial blood pressure of 129/65 mmHg. A 3/6 pansystolic apical murmur was heard by auscultation. Neurological examination revealed neck stiffness. On day two after admission, digital subtraction angiography (DSA) was performed for evaluation of intracerebral aneurysm. But no aneurysm or cerebral vascular malformation was found on the initial DSA (Figures 1D, 1E). Results of the laboratory test were unremarkable except for a slightly elevated white blood cell count (11.9 * 10^9^/L). The electrocardiography (ECG) showed nodal tachycardia. Brain MRI performed on day four revealed a previous infarction in the left frontal lobe (Figures 1F, 1G). Transthoracic echocardiography (TTE) showed a dilated heart and detected a left atrial mass measuring 5.09 * 3.34 cm in size attached to the lower part of interatrial septum, oscillating with heat contraction (Figure 2A). Moderate-to-severe mitral regurgitation and mild tricuspid regurgitation were observed too (Figure 2B). A diagnosis of left atrial myxoma was considered.

The patient was transferred to the Department of Cardiac Surgery and underwent atrial tumor resection soon. A gray, polypoid, colloid-like left atrial mass of 6 * 5 * 2.4 cm in size was excised. No residual tumor tissue was detected by transesophageal echocardiogram (TEE). Pathological examination confirmed the diagnosis of atrial myxoma (Figure 2C). The patient’s symptoms improved in the following two weeks. On day 24 after onset, brain CT angiography (CTA) was performed in order to re-evaluate intracerebral aneurysm, but there was no aneurysm or any other vascular malformation found (Figure 1H). On day 28, the patient was discharged with no residual neurological symptoms.

**Figure 3:**
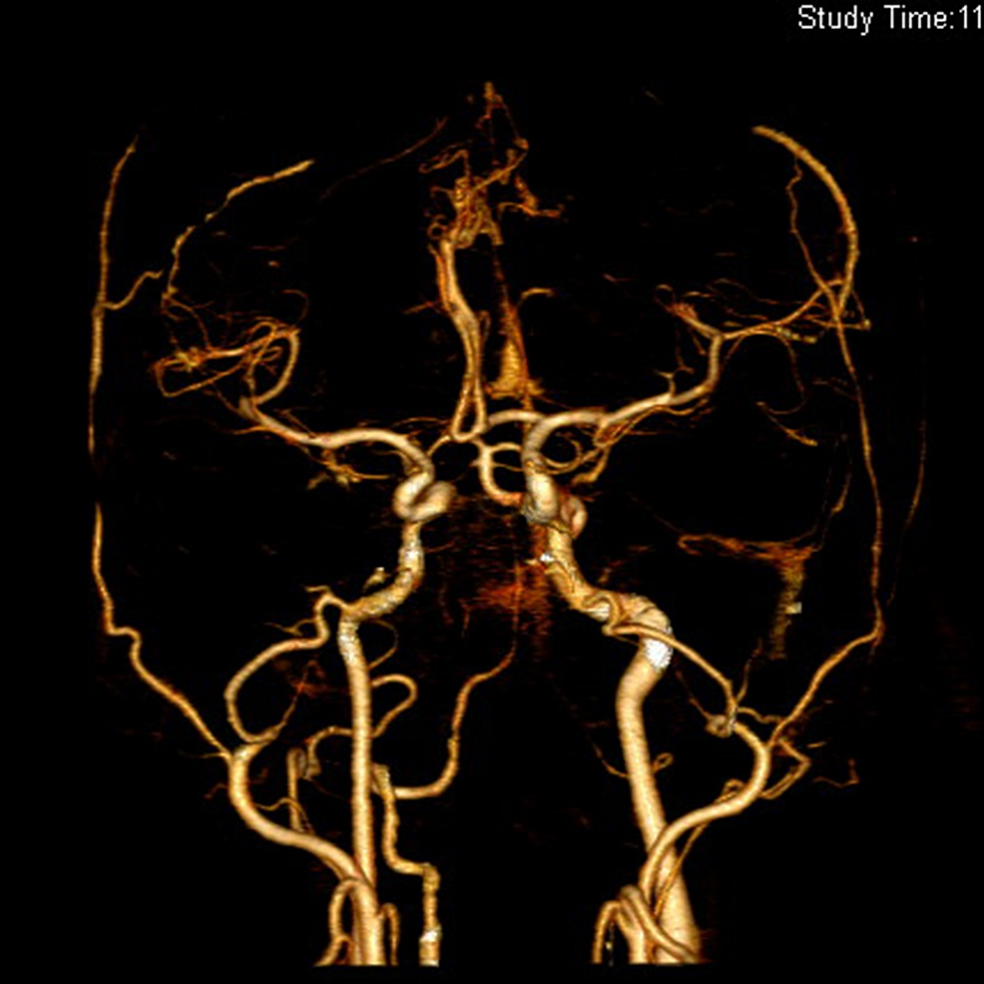
Cerebral CTA performed one year after discharge revealed no aneurysm CTA: computed tomographic angiography

We have followed the patient for one year. He remains asymptomatic since discharge. At six months after discharge, TTE was performed revealing no recurrence of the cardiac tumor. And at 12 months, no abnormality was found by a repeated brain CTA (Figure 3).

## Discussion

In this case, SAH is believed to be associated with left atrial myxoma. The diagnosis was made based on the following reasons. Firstly, previous research has demonstrated that embolic myxoma cells could cause intracerebral hemorrhage via direct vessel invasion or rupture of the myxomatous aneurysm [10,11]. Secondly, the preceded ischemic stroke provided evidence for cerebral embolism by myxoma cells and the possibility of subsequent cerebral vascular damage. Thirdly, other risk factors for SAH and ischemic stroke, such as old age, hypertension, cerebral vascular malformation, brain trauma, diabetes mellitus, or related hereditary trait, are absent in the patient.

Intracerebral hemorrhage, including parenchymal hemorrhage and SAH, following cerebral embolism, is the second common neurological complication of cardiac myxoma [2]. It can occur before or years after diagnosis and resection of cardiac myxoma, usually in the context of preceded cerebral embolism [12]. Embolic myxoma cells can invade vessel walls and damage the internal elastic lamina of brain arteries, leading to abnormal subintimal growth and subsequent aneurysm formation [10,11]. Rupture of damaged cerebral vessels or myxomatous aneurysms ultimately contributes to intracerebral hemorrhage. The true incidence of intracerebral hemorrhage in patients with cardiac myxoma is unclear. It is noted that in most of the previous reports, parenchymal hematoma rather than SAH was observed, despite that cerebral aneurysms were not uncommon in patients with cardiac myxoma [5-9,11,13]. This may be attributed to the common location of myxomatous aneurysms being at the distal branches of cerebral arteries, which is different from that of non-myxomatous aneurysms. In our report, the brain CT scan indicates pattern of aneurysmal SAH, but neither the DSA nor repeated CTA revealed any aneurysm. A follow-up CTA performed one year after SAH reveals no abnormalities. As far as the literatures show, this may be the first case report of angiographic-negative SAH in a patient with cardiac myxoma. One explanation for the negative angiographic result is that the causative aneurysm may be small in size that could not be identified by DSA or CTA. It was found that aneurysms smaller than 3 mm in size were not reliably detected by CTA, with sensitivity of only 40-90% [14,15]. Although DSA has been demonstrated to have more satisfactory sensitivity for evaluating intracerebral aneurysm, about 20-25% of aneurysmal SAH were DSA-negative on initial angiography, and delayed repeated DSA would reveal small aneurysms in 14% of the patients [15]. This explanation is in accord with the typical pattern of myxomatous aneurysms being small in size. The other possibility is that myxoma cells can cause the rupture of embolized cerebral arteries without aneurysm formation. Non-aneurysmal SAH accounts for 15% of all SAH patients, which can be contributed by a variety of injuring factors to major vessels of the brain, such as vasculitis, trauma, amyloid angiopathy, or thrombosis [16]. Although most cardiac myxomas are benign tumors, the embolized myxoma cells can infiltrate into or even through the brain vessels, leading to weakening and rupture of affected arteries. This pathological process may precede the formation of the intracerebral aneurysm and lead to SAH in our reported patient.

Cerebral hemorrhage is a relatively uncommon complication of cardiac myxoma. Although even rarer, SAH may lead to much worse outcomes than cerebral embolism and parenchymal hemorrhage due to higher mortality and morbidity, such as vasospasm, hydrocephalus, and rebleeding. Nevertheless, the result of our report provides evidence that patients with cardiac myxoma can suffer from SAH early in the disease, even in the absence of intracerebral aneurysm. This further suggests the potential risk of hemorrhagic event in patients with embolic complication by myxoma who is otherwise indicated for thrombolytic or anticoagulant treatment. Surgical resection of the tumor is believed to be the only curative treatment for cardiac myxoma and is recommended that it should be applied timely when cardiac myxoma is diagnosed. But for those complicated with cerebral embolism, whether thrombolysis and anticoagulant are safe remains controversial. Some have reported that intravenous thrombolysis using recombinant tissue plasminogen activator (rtPA) is safe and effective for cerebral embolism associated with cardiac myxoma, whereas serious hemorrhagic side effects were seen in others [17-19]. Considering the rupture-prone feature of cerebral vessels of patient with cardiac myxoma, as was shown in our case, caution of hemorrhagic risk should be raised for using intravenous thrombolysis or anticoagulant in cerebral embolism.

There are three limitations in our case report. Firstly, a single case of the patient is presented in the current report. The clinical features of angiographic-negative SAH associated with cardiac myxoma need to be summarized based on observation of more cases. Secondly, CTA instead of DSA was used for the second and follow-up evaluation of intracerebral aneurysm, which may lead to false-negative angiographic findings, due to most myxomatous aneurysms being small size and located in the distal branch of cerebral arteries. Thirdly, we have followed the patient for only one year, which may not be long enough to determine the long-term outcome of SAH associated with cardiac myxoma, since delayed aneurysm formation and rupture are not uncommon in such patients.

## Conclusions

We suggest that cardiac myxoma patients complicated with cerebral hemorrhage should have a routine evaluation for intracerebral aneurysm by DSA. The risk of cerebral hemorrhage due to damaged vessel walls should be considered carefully when myxomatous embolism is otherwise indicated for thrombolytic or anticoagulant treatment. Future studies can use high-resolution MRI to observe the detail of pathological alteration of affected arteries in myxoma patients.
